# Case Report: A Case With MOGAD and Anti-NMDAR Encephalitis Overlapping Syndrome Mimicing Radiological Characteristics of CLIPPERS

**DOI:** 10.3389/fimmu.2022.832084

**Published:** 2022-04-14

**Authors:** Jia Guo, Yujie Bu, Wei Liu

**Affiliations:** Department of Neurology, Lanzhou University Second Hospital, Lanzhou, China

**Keywords:** CLIPPERS mimics, MOGAD, anti-NMDAR encephalitis, MNOS, autoimmune

## Abstract

Herein, we reported a case of a young man diagnosed with MNOS (anti-myelin oligodendrocyte glycoprotein associated disease (MOGAD) and anti-N-methyl-D-aspartate receptor (NMDAR) encephalitis overlapping syndrome, i.e., MNOS), whose imaging findings in magnetic resonance imaging (MRI) mimicked chronic lymphocytic inflammation with pontine perivascular enhancement responsive to steroids (CLIPPERS). We reported a case of refractory anti-NMDAR encephalitis that recurred after standard first-line and second-line treatment. The patient presented with CLIPPERS on imaging at recent hospital admission, and his MOG antibodies were seropositive. After intravenous methylprednisolone (IVMP) treatment, the patient’s symptoms were significantly alleviated. In this case, we demonstrated that MNOS could mimic the radiological characteristics of CLIPPERS. Future studies should focus on the diagnosis and treatment of antibody overlap syndrome.

## Introduction

Chronic lymphocytic inflammation with pontine perivascular enhancement responsive to steroids (CLIPPERS) is a chronic central nervous system inflammatory disorder (1), mainly characterized by lymphocyte infiltration in pons, midbrain, and pericerebrovascular area. It is sensitive to steroid treatment and can also affect other parts of the spinal cord. The disease usually involves both white and deep grey matter, while the cortex is spared. Its imaging diagnosis is mainly based on an enhanced MRI scan of the head, which reveals typical “pepper and salt” enhancement in pons and cerebellum, measuring < 3mm in diameter (2).

MOGAD is a well-described demyelinating disorder with age-dependent clinical features (3) and a broad spectrum of manifestations including optic neuritis (ON), myelitis, brainstem encephalitis, encephalopathy, acute disseminated encephalomyelitis (ADEM), or ADEM-like presentations. Over recent years, the discovery of the coexistence of anti-MOG and anti-NMDAR antibodies has led to a new emerging concept, namely MOGAD and anti-NMDAR encephalitis overlapping syndrome (MNOS) (4–6). MNOS has a relatively high recurrence rate. However, it is sensitive to first-line therapy, and patients tend to have good clinical improvement.

There have been a number of reports about CLIPPERS-mimics. The relationship between CLIPPERS and MOGAD has also received increasing attention (1, 2, 7). Screening MOGAD as a CLIPPERS mimicker is crucial to avoid misdiagnosis (8). Martin et al. had reported a case of anti-NMDAR encephalitis overlapping with CLIPPERS syndrome (9). However, imaging findings of CLIPPERS in MNOS have not yet been reported. Herein, we reported a case of refractory anti-NMDAR encephalitis that recurred after standard first-line and second-line treatments. On the most recent admission, the patient presented with CLIPPERS by imaging and seropositive MOG antibodies. After IVMP treatment, the patient’s symptoms significantly improved.

## Case Presentation

A 25-year-old male complained of fever and headache lasting for 10 days in July 2016. His highest body temperature reached 39°C, and he developed agitation, delirium, orofacial involuntary movements, and seizures, with a of consciousness for 10 min. Brain MRI and electroencephalogram (EEG) were normal. A cerebrospinal fluid (CSF) examination revealed an elevated leucocyte count (110 * 106/L; 84% mononuclear) and a reduced chlorine concentration (116.6mmol/L). Glucose and protein levels were within the normal range. NMDAR-ab titer was 1:10 in CSF, with and negative result in serum. High-dose pulse IVMP and intravenous immunoglobulin (IVIG) regimen (0.4 g/kg/day) were initiated, and patient’s symptoms were gradually relieved, while short-term memory impairment persisted. After discharge, the patient received oral prednisone, (titrated to 50mg/day) and mycofenolate mofetil (MMF) (1000 mg/d). One month later, NMDAR-ab titer in CSF decreased to 1:1.

**Figure 1:**
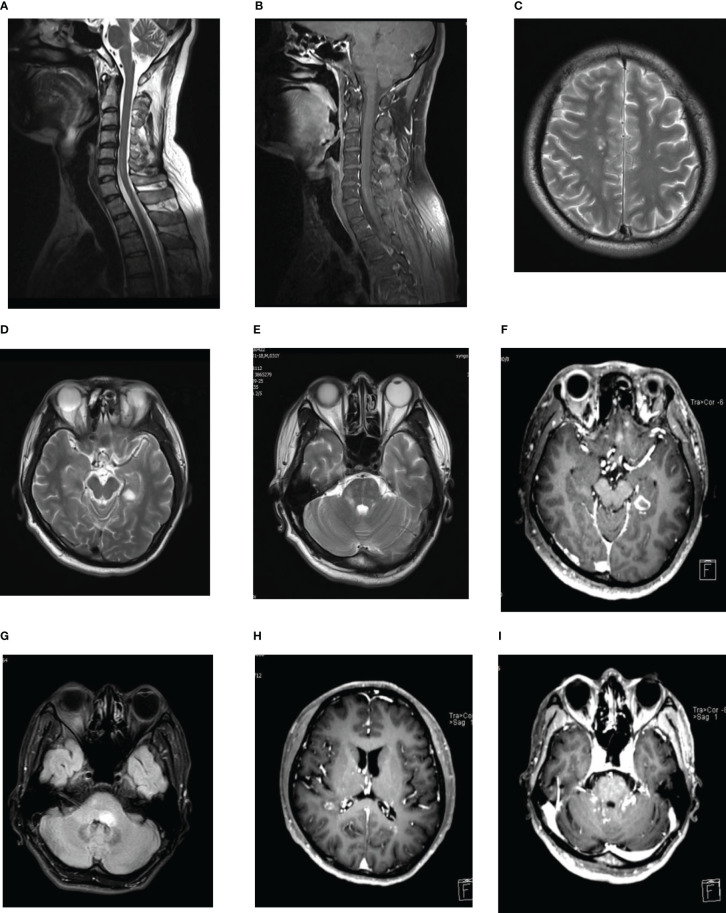
**(A, B)** Spinal cord MRI showed T2-hypertense lesions at C5-6 **(A)** with slight Gd enhancement **(B)**. **(C–F)** Multiple lesions on MRI of the brain. T2-hypertense in right center semicovale, lateral paraventricular, pons, left temporal lobe, with an obvious contrast-enhancing lesions in the left temporal lobe. **(G)** Brain MRI revealed a new T2-hyperintense lesion located in left pons with no-contrast enhancement. **(H, I)** Gd-enhanced MRI showed punctate and curvilinear enhancing lesions involving the right semi-oval center areas, the anterior and posterior angles of lateral ventricle, pons and bilateral cerebellar hemisphere in the right hemispheres of hemispheres, the anterior and posterior angles of lateral ventricles, pons and left cerebellar hemispheres, without evidence of leptomeningeal enhancement.

Six months later, the patient developed diplopia and unsteady walking. Neurological examination revealed bilateral abduction limitation, nystagmus, and bilateral Hoffman sign. Brain MRI showed no obvious abnormalities and no Gd enhancement. Spinal cord MRI showed T2-hypertense lesions at C5-6 with slight Gd enhancement (**Figures 1A, B**). Repeated NMDAR-ab examinations in CSF and serum showed a titer of1:32 and 1:10, respectively. Meanwhile, serum anti-MOG and anti-Aquaporin4 (AQP4) antibodies tests revealed negative results, and no oligoclonal bands (OB) were detected. The patient received IVIG (0.4 g/kg/d) injection for 5 days. However, no significant improvement was observed. One month later, positive NMDAR-ab antibodies in CSF (titer: 1:320) and negative test in serum were observed. In addition, OB, serum anti-MOG, anti-AQP4, and paraneoplastic antibody tests all showed negative results. After treatment with IVMP (1000mg per day for 5 days), the patient’s symptoms improved. He was diagnosed with anti-NMDAR encephalitis involving the spinal cord. To prevent relapse, he was given oral prednisone (50 mg/day, regularly taper the steroid dose) and MMF (1500 mg/d) after discharge.

In September 2017, the patient presented with blurred vision, right limb weakness, and numbness in the right side of the body. Later, the patient gradually developed headaches, diplopia, unsteadiness while walking, and numbness of the left finger. Neurological examination showed decreased visual acuity, right limb weakness, and bilateral Babinski signs. Brain MRI showed multiple lesions in the right-center semicovale, lateral paraventricular, pons and left temporal lobe, and obvious contrast-enhancing lesions in the left temporal lobe (**Figures 1C–F**). Spinal cord MRI and orbital MR were normal. Visual evoked potentials showed significantly delayed P100 latency in the left eye, and no definite wave was elicited in right eye. Optical coherence tomography showed retinal nerve fiber layer thickness thinning in both eyes. Positive NMDAR antibodies were detected in both serum and CSF (titer: 1:32). Repeated examinations of serum MOG and AQP4 antibodies, and OB all showed unremarkable results. Combined with previous clinical findings, the patient was diagnosed with anti-NMDAR encephalitis accompanied by ON and myelitis, which didn’t fulfill the criteria for serum-negative neuromyelitis optica spectrum disorder (NMOSD). After treatments with IVMP and IVIG, the patient’s symptoms improved. Rituximab (100mg) was sequentially administered every six months to prevent a recurrence. Considering the possible side-effects associated with taking both immunosuppressants at the same time, oral prednisone and MMF were gradually reduced. Follow-up MRI revealed significantly diminished lesions, and the patient’s condition remained stable.

In May 2020, the patient readmitted for left peripheral facial palsy. Brain MRI revealed a new T2-hyperintense lesion in the left pons with no-contrast enhancement (**Figure 1G**). CSF test showed 19 * 106/L white blood cells (100% mononuclear), normal protein levels, and Gram-stain-negativity. CSF NMDAR antibody testing showed a positive result (titer: 1:1), whereas negative serum NMDAR, MOG and AQP4 antibodies were indicated. Before this admission, the patient had been treated with 160 mg/d methylprednisolone in a local hospital for peripheral facial palsy. Having previously experienced severe skin rash and tremor side-effects after using steroids, the patient did not agree to increase the dose of steroids. After treatment with IVMP (120 mg/d for 3 days, 80 mg/d for 3 days, and 40 mg/d for 3 days), his symptoms were again relieved. As the disease had progressed despite a long period of regular rituximab infusion, and the patient refused to use steroids again, he continued to take MMF (1500mg/d) after discharge with rituximab infusion at regular intervals, and the lymphocyte subset (B-cell) counting was regularly performed. The results suggested satisfactory B-cell depletion. Four months later, follow-up MRI revealed that pons lesions disappeared.

In June 2021, the patient developed vertigo, slurred speech, and ataxia. Brain MRI was performed at a local hospital, and the results showed abnormal signals in bilateral lateral ventricles and cerebellum. After admission, gadolinium-enhanced MRI showed punctate and curvilinear enhancing lesions involving the right semi-oval center areas, anterior and posterior horns of lateral ventricles, pons and bilateral cerebellum in the right hemisphere, the anterior and posterior horns of lateral ventricles, pons and left cerebellar hemisphere, without evidence of leptomeningeal enhancement (**Figures 1H, I**). These findings were consistent with the radiological manifestations of CLIPPERS (2). CSF test showed 47* 106/L white blood cells (95% mononuclear), with normal protein level, and Gram-stain-negative. Negative serum NMDAR antibodies and positive results in cerebrospinal fluid (titer: 1:1) were found. Based on the patient’s clinical manifestations, imaging findings and CSF biochemical results, demyelinating disorders were considered first. Additionally, positive serum MOG antibodies (titer: 1:10), and negative serum AQP4 and Glial fibrillary acidic protein (GFAP) antibodies, and OB were revealed. So, we did not further perform the metagenomic next-generation sequencing of CSF to exclude infection. However, with regularly rituximab infusion, the patient reported new symptoms again, with a serum-positive MOG antibody result. MOGAD is highly sensitive to steroids. After communicating with the patient, we decided to discontinue rituximab, administer high-dose methylprednisolone pulse therapy. After treatment with IVMP (1000mg/d for 3days, and 500mg/d for 3days), the patient’s symptoms improved, and he continued taking oral prednisone and MMF.

**Figure 2:**
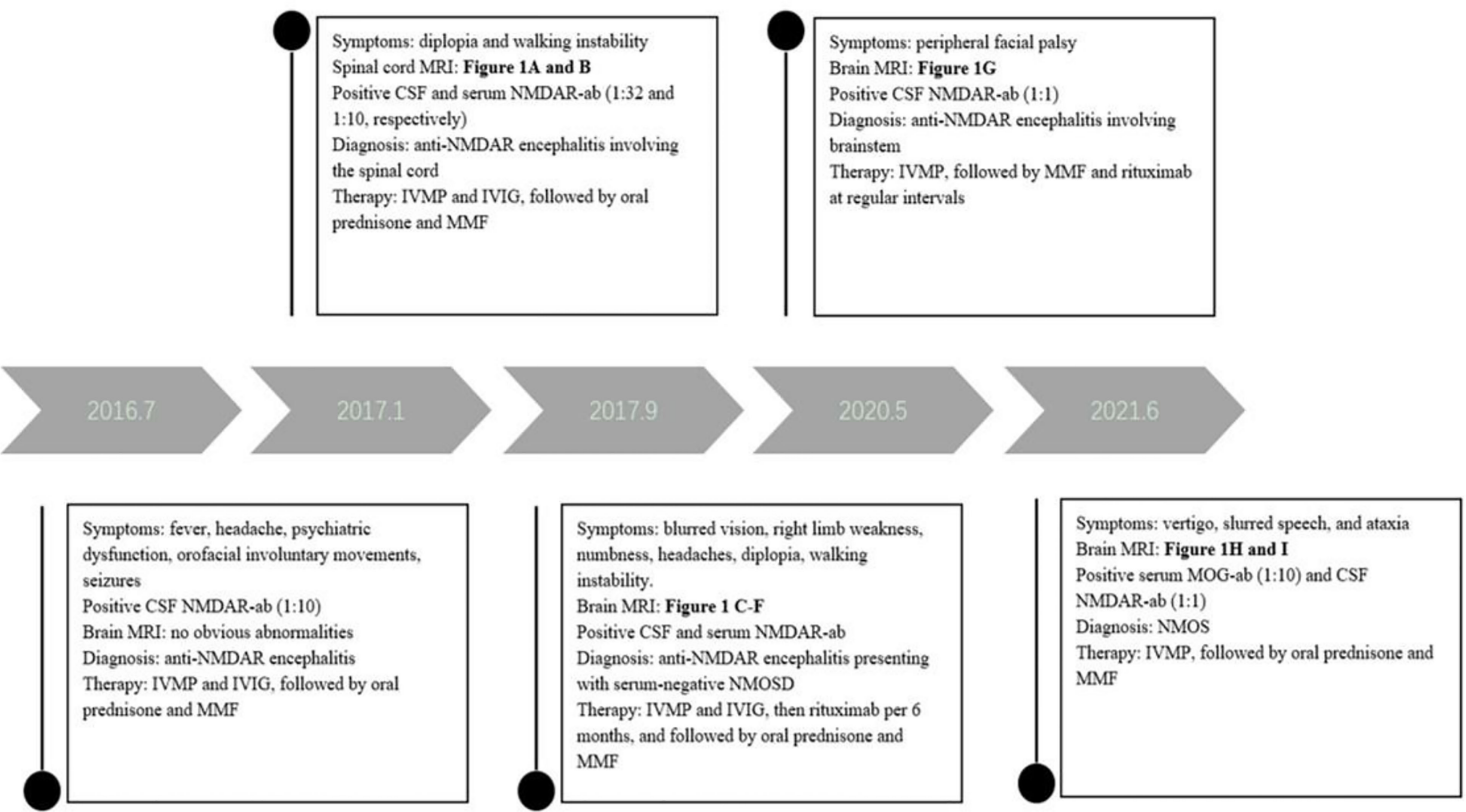
The timeline of relevant results and interventions during the diagnosis and treatment of our patient.

The patient underwent tumor screening during several admissions, including tumor marker screening, and ultrasound and computed tomography examinations. No tumor has been found so far. Multiple examinations on NMDAR, MOG, AQP4 and GFAP antibodies, and OB were all performed by the Chinese branch of the Euroimmun Medical Diagnostic Laboratory using a fixed cell-based indirect immune-fluorescence test (IIFT) (Euroimmun, Germany). The relevant diagnosis and treatment results of the patient are showed in **Figure 2**.

## Discussion

It has been suggested that anti-NMDAR encephalitis may be accompanied by concurrent or separate episodes of demyelinating disorders, such as MOGAD or NMOSD (10). Anti-NMDAR encephalitis is often more difficult to treat when it coexists with other demyelinating diseases (10). Herein, we reported a case of MNOS. Antibody co-existence is possible, for example, the antigens of MOG and NMDA can simultaneously coexist on the surface of oligodendrocytes (11). However, the mechanism underlying this phenomenon remains unclear and may be related to immune reconstitution during disease development and treatment (12). Unlike common anti-NMDAR encephalitis, MNOS is unlikely associated with tumors. Moreover, the titer of antibodies is often not proportional to the severity of the disease (13).

The patient, in this study initially had a diagnosis of anti-NMDAR encephalitis. After repeated first-line therapy (including high-dose steroids and IVIG), the patient’s autoimmune encephalitis symptoms were stable after final adjustment to low-dose rituximab. During follow-up, NMDAR antibody titer in the patient’s CSF fluctuated between negative value and 1:1. The patient presented with decreased visual acuity and two episodes of brainstem symptoms, which were considered to be associated with demyelinating disease. However, serum levels of central nervous system demyelination-associated antibodies (MOG, AQP4, and GFAP) using standard tests consistently showed negative values over the previous disease course. OB has also never been detected either. It has been reported that anti-NMDAR encephalitis can be accompanied by demyelination episodes, presenting atypical symptoms of anti-NMDAR encephalitis, while the antibodies associated with demyelination symptoms (such as those against AQP4 or MOG) have not been detected (10, 14). Therefore, the patient was initially considered to have atypical anti-NMDAR encephalitis with ON and myelitis. Yet, MOG antibodies detected in serum only at the most recent episode after a lapse of 4 years, which is uncommon for MNOS. Due to the low-titer MOG antibody result, we did consider the possibility of a false positive. However, studies have shown that antibody titers may be low due to long-term immunosuppressive therapy (15, 16). Considering the patient’s several imaging findings, we believe that the positive MOG antibody result was pathogenic. The patient later chose rituximab as the second-line therapy for anti-NMDAR encephalitis, which resulted in satisfactory B-cell depletion. The patient’s clinical performance was improved, and the NMDAR antibody titer did not significantly increase. MOGAD is highly sensitive to steroids. The optimal treatment for reducing relapses of MOGAD is still not determined. Current guidelines recommend rituximab as the second-line treatment or later maintenance therapy for MOGAD (17). However, a large retrospective multicenter study suggested that 61% of MOGAD patients experienced relapse whilst on rituximab maintenance (18). Whittam et al. also showed that rituximab reduced relapse rates in MOGAD. Many patients still continued to experience relapse despite apparent B-cell depletion (19). It is possible that the diagnosis and treatment of the patient in this case report also indirectly reflect the poor efficacy of rituximab for MOGAD. After detecting MOG antibodies in serum, the patient was given high-dose steroids, and his symptoms significantly improved. This was consistent with the good prognosis of MNOS reported in previous literature (6).

Many central nervous system diseases, including MOGAD, may mimic CLIPPERS both clinically and radiologically (20), as CLIPPERS may be only pathologically described with a slowly expanding spectrum of specific etiological disorders. The importance and necessity of screening for MOG antibodies in patients with clinical and radiological features of CLIPPERS has been emphasized. Interestingly, the patient in this case study has been diagnosed with anti-NMDAR encephalitis for four years and showed positive serum MOG antibodies during a recent episode; yet, his radiological findings suggested CLIPPERS characteristics. A few cases of CLIPPERS combined with MOG antibody-positivity have been previously reported, but there was no case of CLIPPERS combined with MNOS. Our case report might possibly enrich the disease spectrum of CLIPPERS-mimics.

## Conclusion

Numerous studies have reported antibody overlap syndrome, revealing that multiple antibody tests may be required when clinical and radiological manifestations are atypical or suggestive of other antibody-related diseases. The treatment and prognosis of different antibody-related diseases vary, which is of great significance for precision therapy. Further studies are warranted to help clarify the possibility that MNOS may increase the spectrum of CLIPPERS.

## Data Availability Statement

The original contributions presented in the study are included in the article/supplementary material. Further inquiries can be directed to the corresponding author.

## Ethics Statement

The studies involving human participants were reviewed and approved by Ethics Committee of the Lanzhou University Second Hospital, Lanzhou. The patients/participants provided their written informed consent to participate in this study. Written informed consent was obtained from the individual(s) for the publication of any potentially identifiable images or data included in this article.

## Author Contributions

JG and YB carried out the studies, participated in collecting data, and drafted the manuscript. JG and WL performed conception and participated in its design. All authors have read the final manuscript and approved it for submission.

## Conflict of Interest

The authors declare that the research was conducted in the absence of any commercial or financial relationships that could be construed as a potential conflict of interest.

## Publisher’s Note

All claims expressed in this article are solely those of the authors and do not necessarily represent those of their affiliated organizations, or those of the publisher, the editors and the reviewers. Any product that may be evaluated in this article, or claim that may be made by its manufacturer, is not guaranteed or endorsed by the publisher.
